# Supplementary material for: Age-associated B cells predict impaired humoral immunity after COVID-19 vaccination in patients receiving immune checkpoint blockade
Source: Nat Commun. 2023 Jun 27;14:3292. doi: 10.1038/s41467-023-38810-0 (PMC10299999; doi:10.1038/s41467-023-38810-0)
Supplement: Supplementary file 1 — Supplementary Information [file 41467_2023_38810_MOESM1_ESM.pdf]

Supplementary Information

Age-Associated B cells predict impaired humoral immunity after COVID-19 vaccination in patients receiving immune checkpoint blockade

Juan Carlos Yam-Puc[1]†, Zhaleh Hosseini[1]†, Emily C. Horner[1]†, Pehuén Pereyra Gerber[2,3]†, Nonantzin Beristain-Covarrubias[1]†, Robert Hughes[1]†, Aleksei Lulla[4], Maria Rust[1], Rebecca Boston[1], Magda Ali[1], Katrin Fischer[4], Edward Simmons-Rosello[1], Martin O'Reilly[1], Harry Robson[1], Lucy H. Booth[1], Lakmini Kahanawita[1], Andrea Correa-Noguera[5], David Favara[5], Lourdes Ceron-Gutierrez[6], Baerbel Keller[7,8], Andrew Craxton[1], Georgina S.F. Anderson[1], Xiao-Ming Sun[1], Anne Elmer[9], Caroline Saunders[9], Areti Bermperi[9], Sherly Jose[9], Nathalie Kingston[10], Thomas E. Mulroney[1], Lucia P. G. Piñon[1], CITIID-NIHR COVID-19 BioResource Collaboration, Michael A. Chapman[1], Sofia Grigoriadou[11], Marion MacFarlane[1], Anne E. Willis[1], Kiran R. Patil[1], Sarah Spencer[1], Emily Staples[1,6], Klaus Warnatz[7,8,12], Matthew S. Buckland[11,13], Florian Hollfelder[4], Marko Hyvönen[4], Rainer Döffinger[6], Christine Parkinson[5], Sara Lear[6], Nicholas J. Matheson[2,3,14]†, James E. D. Thaventhiran[1,6]†.

[1] Medical Research Council Toxicology Unit, School of Biological Sciences, University of Cambridge, Cambridge, UK.

[2] Cambridge Institute of Therapeutic Immunology and Infectious Disease (CITIID), University of Cambridge, Cambridge, UK

[3] Department of Medicine, University of Cambridge, Cambridge, UK

[4] Department of Biochemistry, University of Cambridge, Cambridge, UK

[5] Department of Oncology, Cambridge University NHS Hospitals Foundation Trust, Cambridge, UK

[6] Department of Clinical Immunology, Cambridge University NHS Hospitals Foundation Trust, Cambridge, UK

[7] Department of Rheumatology and Clinical Immunology, Medical Center - University of Freiburg, Faculty of Medicine, University of Freiburg, Freiburg, Germany.

[8] Center for Chronic Immunodeficiency (CCI), Medical Center - University of Freiburg, Faculty of Medicine, University of Freiburg, Freiburg, Germany.

[9] NIHR Cambridge Clinical Research Facility, Cambridge, UK

[10] NIHR BioResource, Cambridge University Hospitals NHS Foundation Trust, Cambridge, UK

[11] Department of Clinical Immunology, Barts Health, London, UK

[12] Department of Immunology, University Hospital Zurich, Zurich, Switzerland

[13] UCL GOSH Institute of Child Health Division of Infection and Immunity, Section of Cellular and Molecular Immunology, London, UK

[14] NHS Blood and Transplant, Cambridge, UK

† These authors contributed equally

* Corresponding authors: jedt2@cam.ac.uk, jcy28@cam.ac.uk.

Supplementary Figure 1.

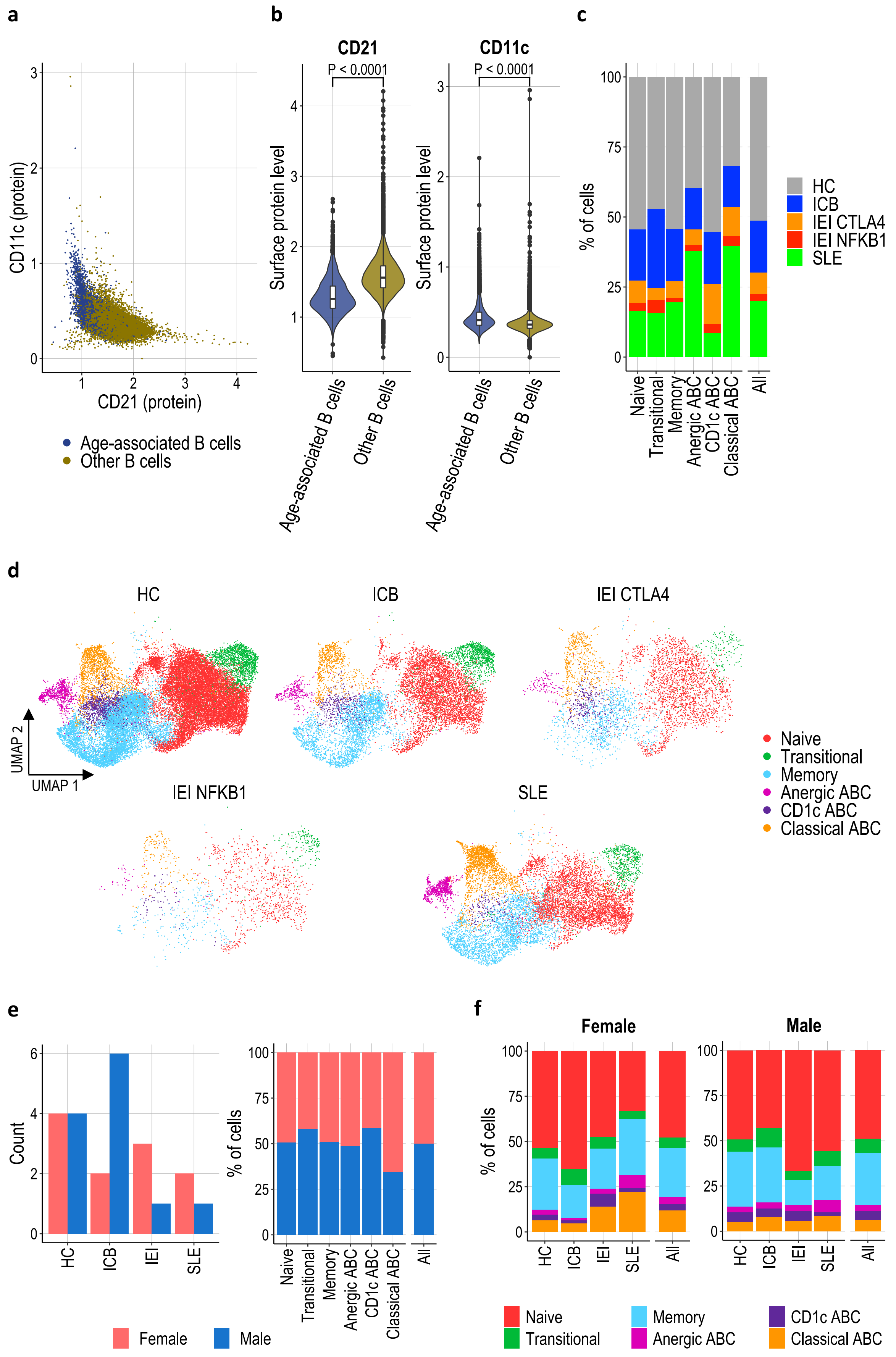

Supplementary Figure 1. Single cell assessment of B cells from different patients. a. Magnetically enriched B cells from PBMCs were labelled using barcode featuring and analysed through droplet-based single cell RNA sequencing technology. Expression of surface protein CD11c against CD21 in all B cells. **b.** Violin plots of CD21 and CD11c surface protein expression in ABCs (n=6048) versus other B cells (n=35948) with CLR-normalised expression values. In the boxplot, the centre, lower and upper bounds of the box correspond to the median, first quartile and third quartile respectively. The upper(lower) whisker extends from the box to the largest(smallest) value no further than 1.5 * IQR from the box upper(lower) bound (where IQR is the inter-quartile range, or distance between the first and third quartiles). Statistical testing via two-tailed Wilcoxon rank sum test with Bonferroni correction. **c.** B cell clusters, with percentages of cells from healthy controls and patients coloured. **d.** UMAPs of total B cells per health condition coloured by annotated clusters (HC, healthy controls; ICB, immune-checkpoint blockade treated patients; IEI CTLA-4/NFKB1, immune errors of immunity, CTLA-4 and NFKB1 mutants respectively; SLE, systemic lupus erythematosus patients). **e.** Number of samples from each gender, coloured as indicated, in healthy controls and patients (Left). B cell clusters, with percentages of cells from each gender coloured (Right). **f.** Healthy controls and patients, with percentages of cells from each cluster coloured as indicated, divided into two groups based on sex. Dots represent a single cell (a-b and d).

Supplementary Figure 2.

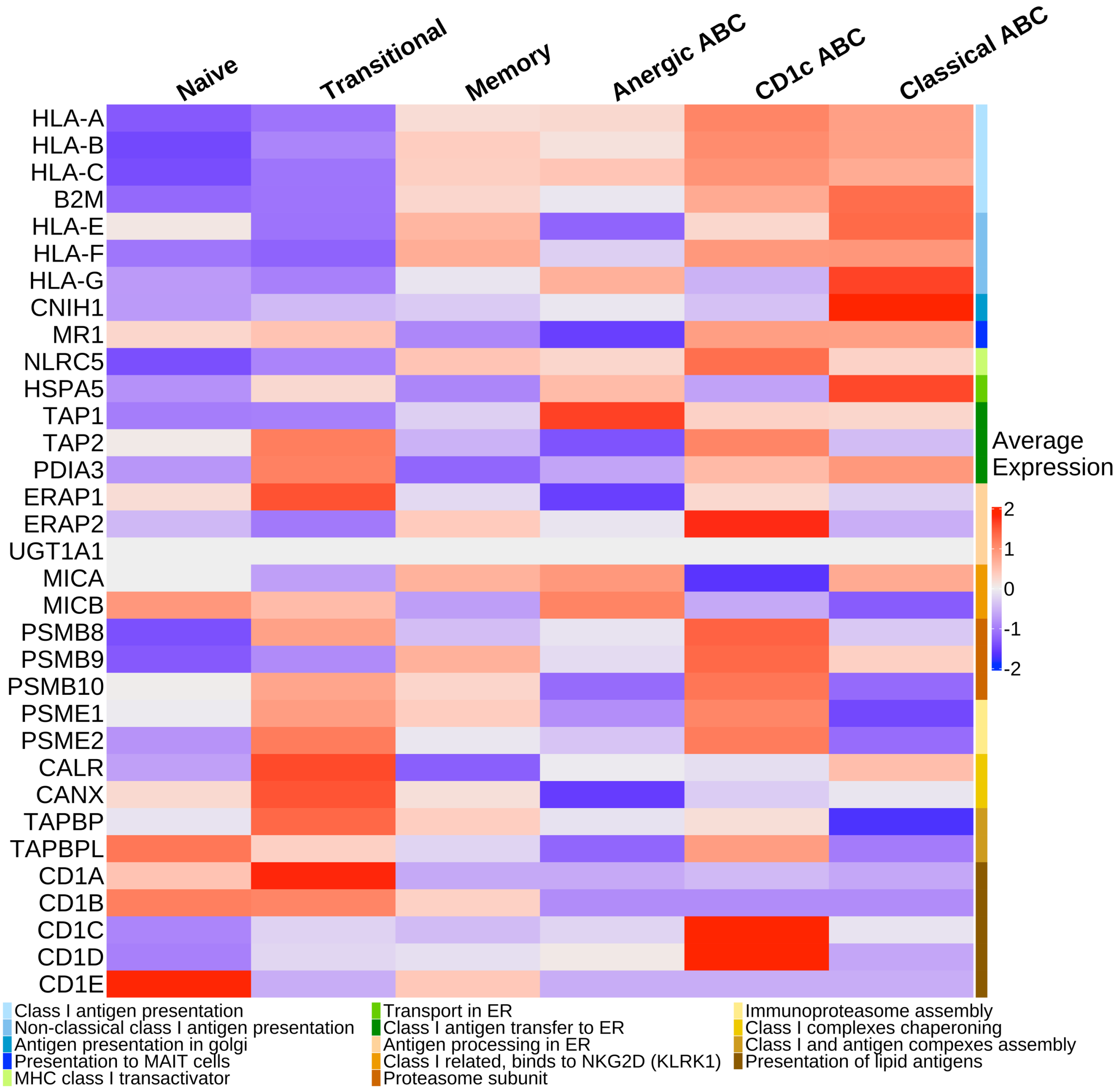

Supplementary Figure 2. Class-I related genes expression within the different B cell clusters. Heatmap representing scaled expression values of genes associated with antigen uptake, processing and class-I presentation in each cluster.

Supplementary Figure 3.

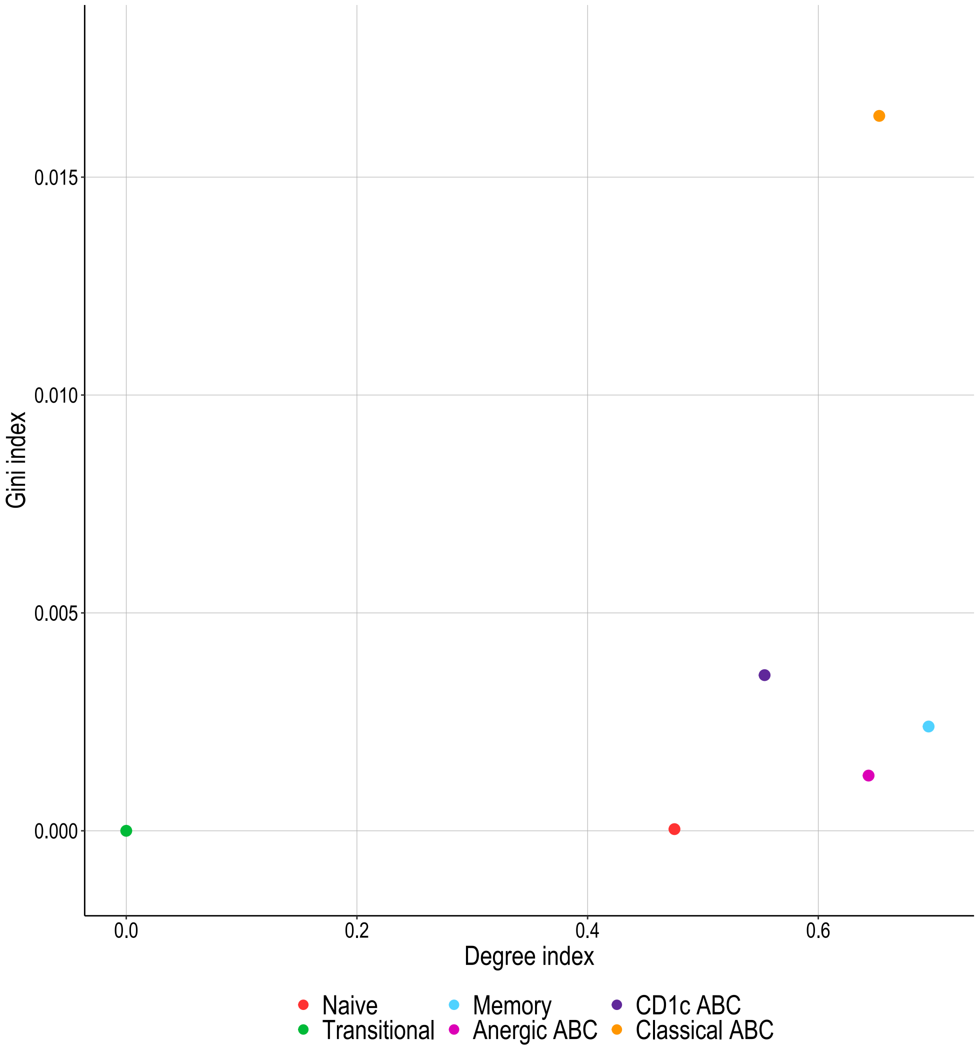

Supplementary Figure 3. Clonal expansion and clonal relationships of different B cell subsets. Dot plot of Gini index against Degree index.

Supplementary Figure 4.

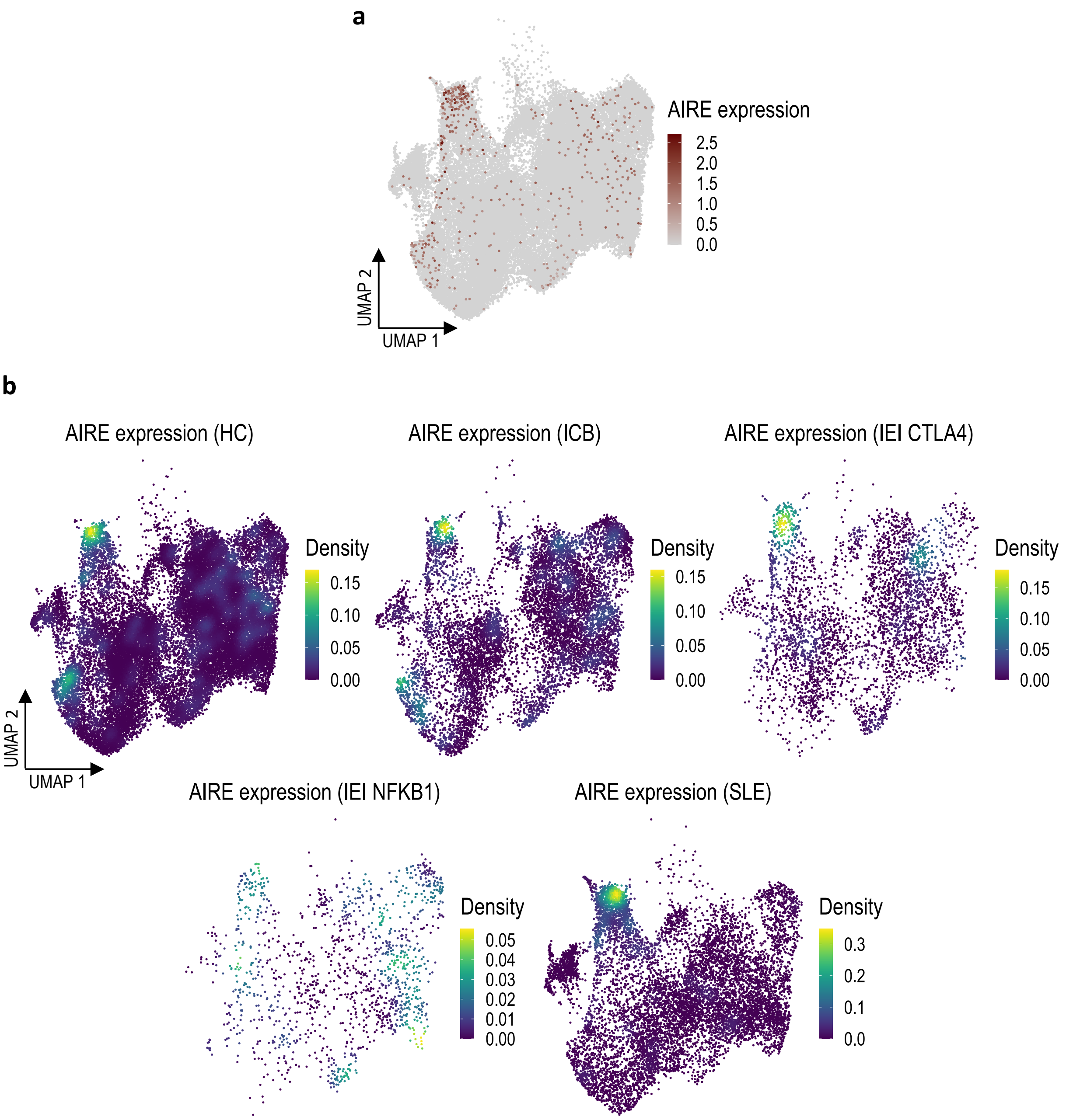

Supplementary Figure 4. Classical age-associated B cells upregulate *AIRE* in different health conditions. a. UMAP of total B cells showing AIRE+ cells coloured by density of expression. **b.** UMAPs of total B cells per health condition coloured by kernel density estimation of *AIRE* expression level (HC, healthy controls; ICB, immune-checkpoint blockade treated patients; IEI CTLA-4/NFKB1, immune errors of immunity, CTLA-4 and NFKB1 mutants respectively; SLE, systemic lupus erythematosus patients).

Supplementary Figure 5.

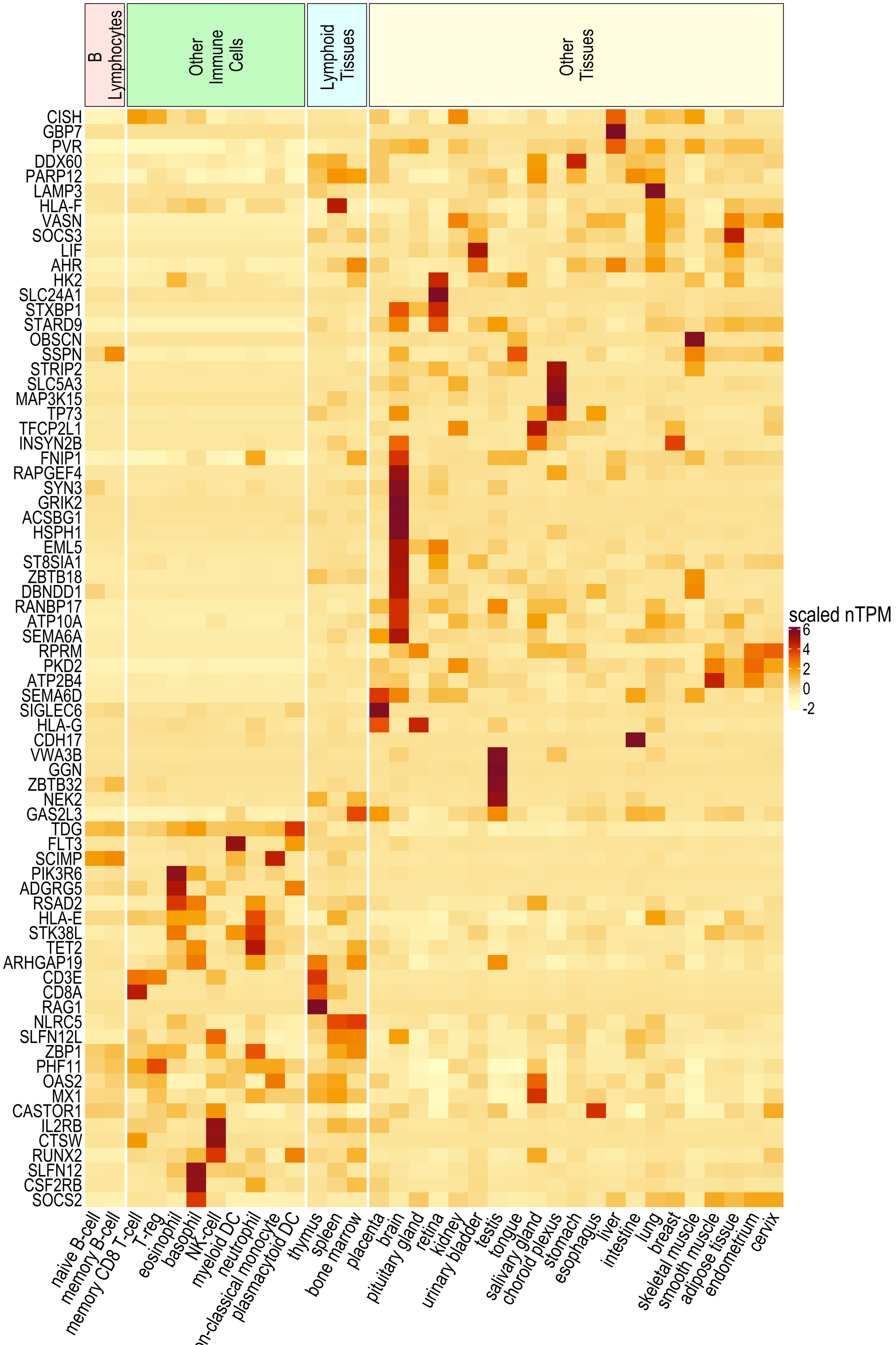

Supplementary Figure 5. Expression of B cell AIRE targets in different cells/tissues. Heatmap representing scaled transcript per million (nTPM) values.

Supplementary Figure 6.

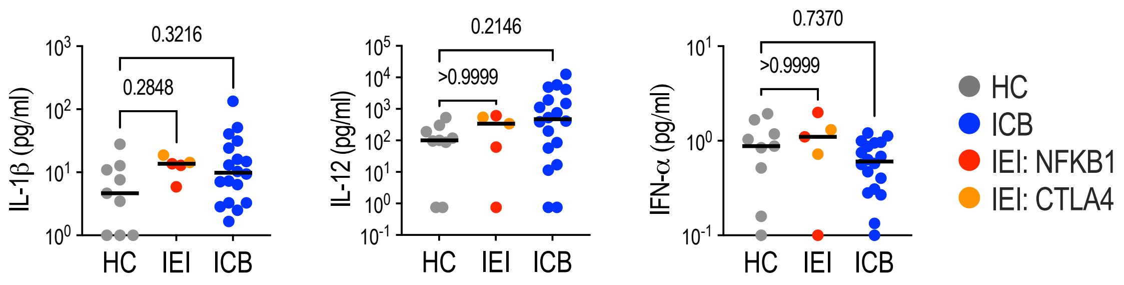

Supplementary Figure 6. Analysis of cytokine levels in serum. Summary graphs showing IL-1β, IL-12 and IFN-α concentrations (pg/ml) in serum from HC=9, healthy controls; IEI=5, immune errors of immunity patients; and ICB=17, immune-checkpoint blockade treated patients 24h after the second dose of BNT162b2 vaccine. Differences between groups were determined using two-tailed non-parametric Kruskal-Wallis tests.

Supplementary Figure 7.

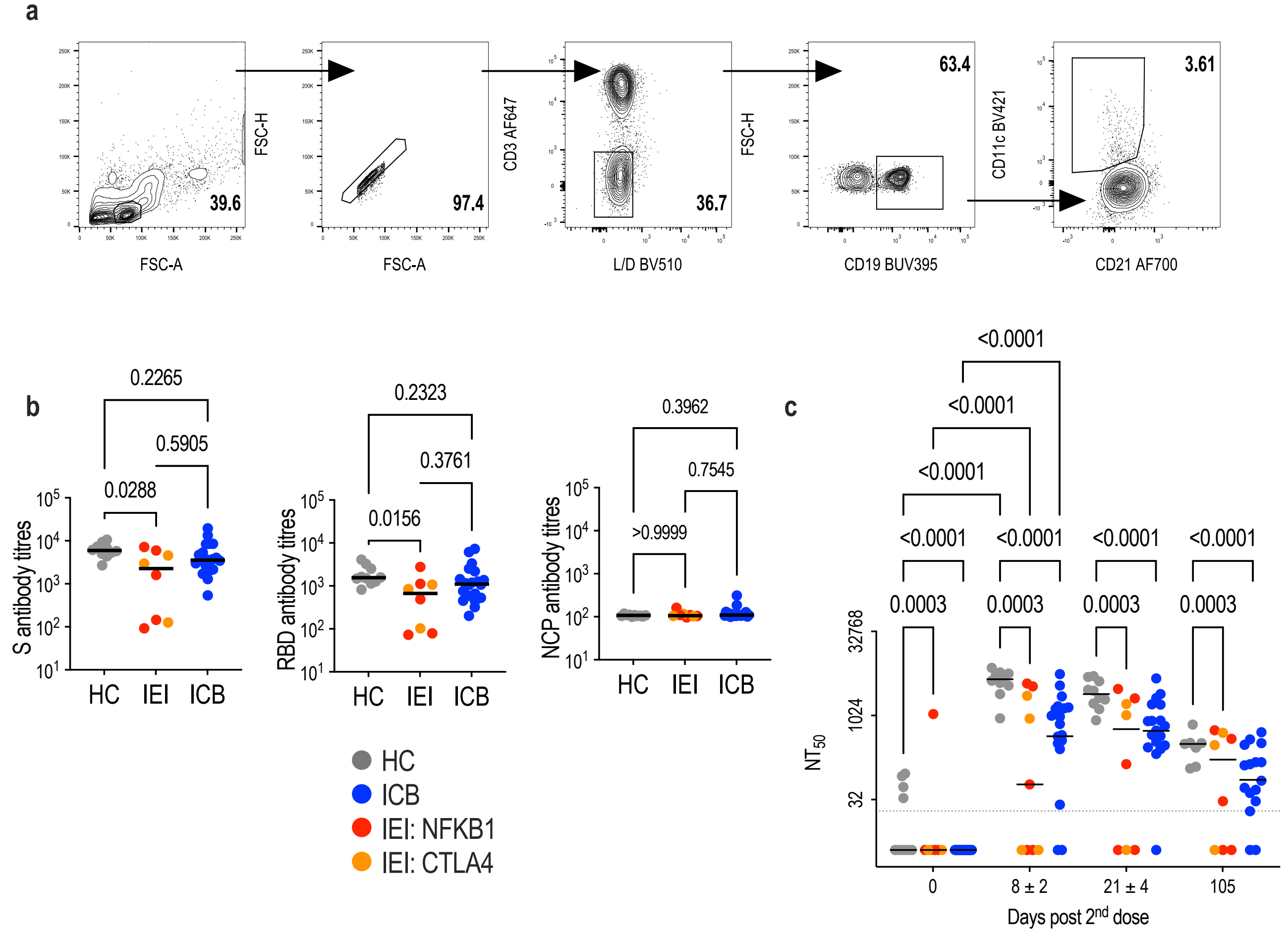

Supplementary Figure 7. Decline of the neutralising capacity after second dose of BNT162b2 vaccine. a. Gating strategy to identify age-associated B cell (ABCs, SingletsLiveCD3-CD19+CD21-CD11+) subset from total B cell population (CD19+). **b.** IgG end-point titres specific to the spike (S), receptor-binding domain (RBD) and nucleocapsid (NCP) proteins of SARS-CoV2 at day 21 after the second dose of BNT162b2 vaccine. HC=10, IEI=8, ICB=19 biologically independent samples. **c**. Neutralising antibody titres at 50% inhibition (NT_{50}) against wildtype SARS-CoV-2 at indicated timepoints after 2nd dose of BNT162b2 vaccine (healthy controls (HC, D0=10, D8=10, D21=10, D105=7), grey; patients with rare inborn errors of immunity (IEI, D0=8, D8=9, D21=8, D105=8): (NFKB1) red, (CTLA-4 and unclassified) orange; patients treated with ICB (ICB, D0=19, D8=19, D21=18, D105=16), blue). The limit of detection of the assays is shown (grey dotted line at NT_{50} = 20). Where specified, statistical significance between groups was determined using 2-way ANOVA with Tukey's multiple comparisons test.

Supplementary Figure 8.

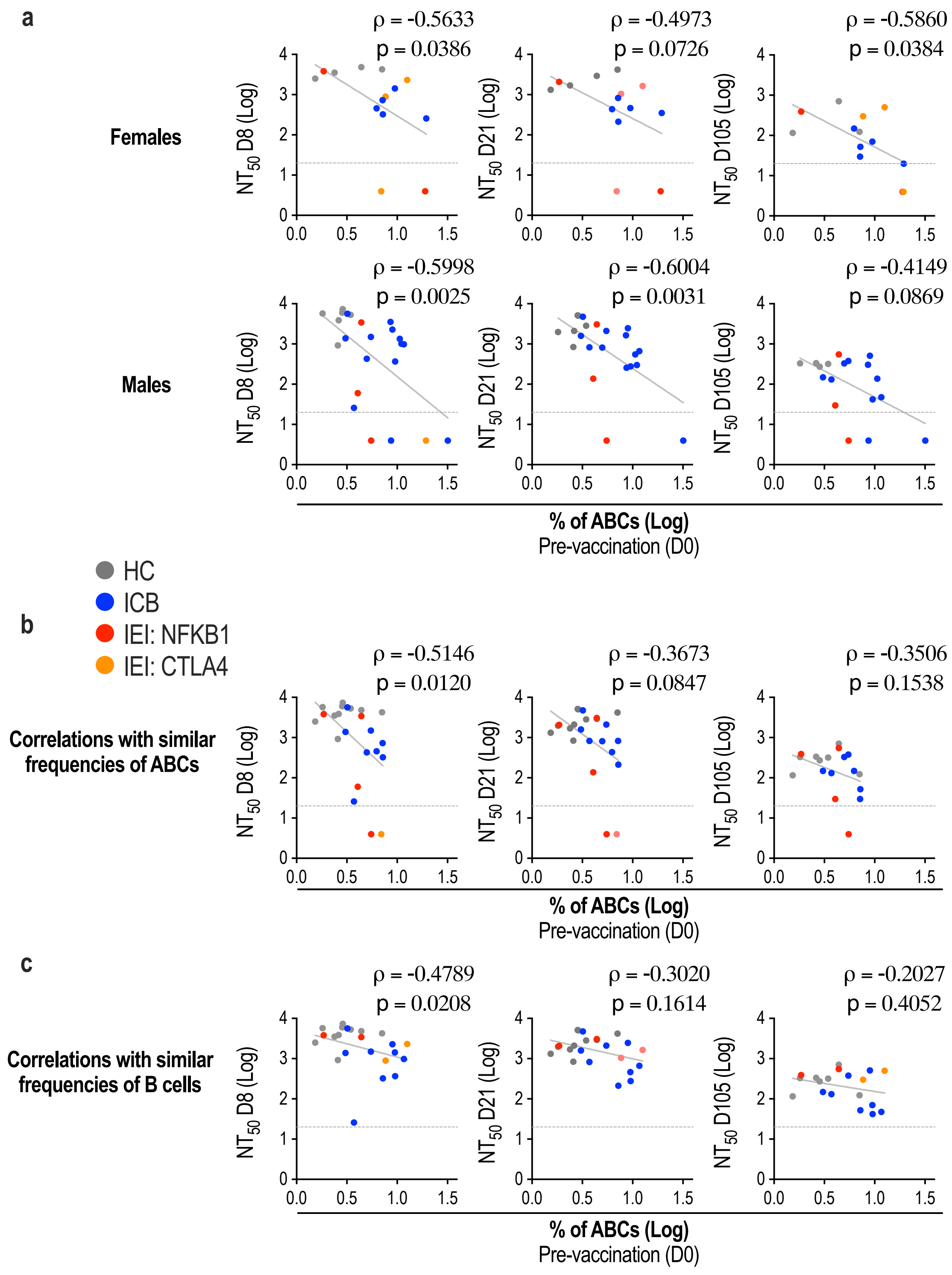

Supplementary Figure 8. Correlations of ABC frequency and neutralising capacity. a. Spearman's correlations of ABC frequency at day 0 and NT_{50} at days 8, 21 and 105 in males and females. Two-tailed Spearman's rank correlation coefficients (rho) and p values are shown, together with indicative linear regression lines. **b.** Spearman's correlations of ABC frequency at day 0 and NT_{50} at days 8, 21 and 105 in the subset of the cohort which had an ABC frequency within the range of healthy controls. Two-tailed Spearman's rank correlation coefficients (rho) and p values are shown, together with indicative linear regression lines. **c.** Spearman's correlations of ABC frequency at day 0 and NT_{50} at days 8, 21 and 105 in the subset of the cohort which had a B lymnphocyte frequency within the range of healthy controls. Two-tailed Spearman's rank correlation coefficients (rho) and p values are shown, together with indicative linear regression lines.

Supplementary Figure 9.

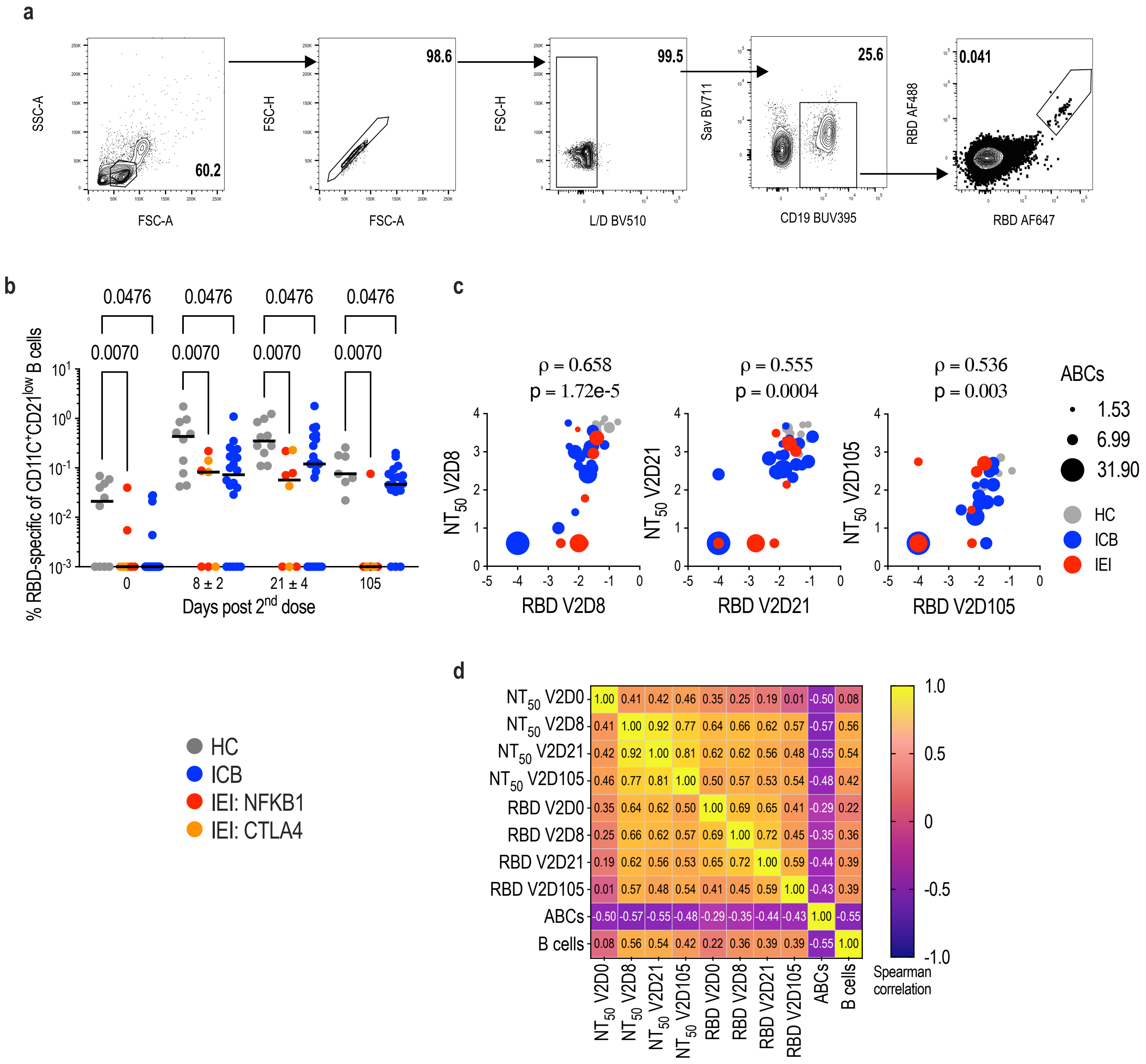

Supplementary Figure 9. ABC expansion is associated with impaired humoral responses to COVID-19 vaccination. a. Gating strategy to identify RBD-specific B cell (SingletsLiveStreptavidinBV711-CD19+RBDAF488+RBDAF647+) subset after the second dose of BNT162b2 vaccine. **b.** Frequencies of RBD-specific ABCs amongst all ABCs ($CD21^{lo}CD11c^{+}CD19^{+}$ B cells) in healthy controls and patients at days 0, 8, 21 and 105. Each dot represents a single individual (healthy controls (HC, D0=10, D8=10, D21=10, D105=7), grey; patients with rare inborn errors of immunity (IEI, D0=7, D8=7, D21=8, D21=6): (NFKB1) red, (CTLA-4 and unclassified) orange; patients treated with ICB (ICB, D0=19, D8=19, D21=19, D105=17), blue). Two-way ANOVA with Tukey's multiple comparisons test for statistical analysis of RBD-binding B cells amongst groups. P values are indicated. **c.** Multiple variable dot plots showing Spearman correlations between RBD-specific B cells and NT_{50} at days 8, 21 and 105 after the second dose of BNT162b2 vaccine. Dot size represents ABC frequency per individual. Colours indicate healthy controls and patients. Two-tailed Spearman's rank correlation coefficients (rho) and p values are shown, together with indicative linear regression line. **d.** Correlation matrix using Spearman's rank correlation amongst ABC and B cell frequencies at day 0; and RBD frequencies and NT_{50} values at days 0, 8, 21 and 105 for the second dose of BNT162b2 vaccine. Data include all samples enrolled in the study; Rho values are shown per correlation.

Supplementary Figure 10.

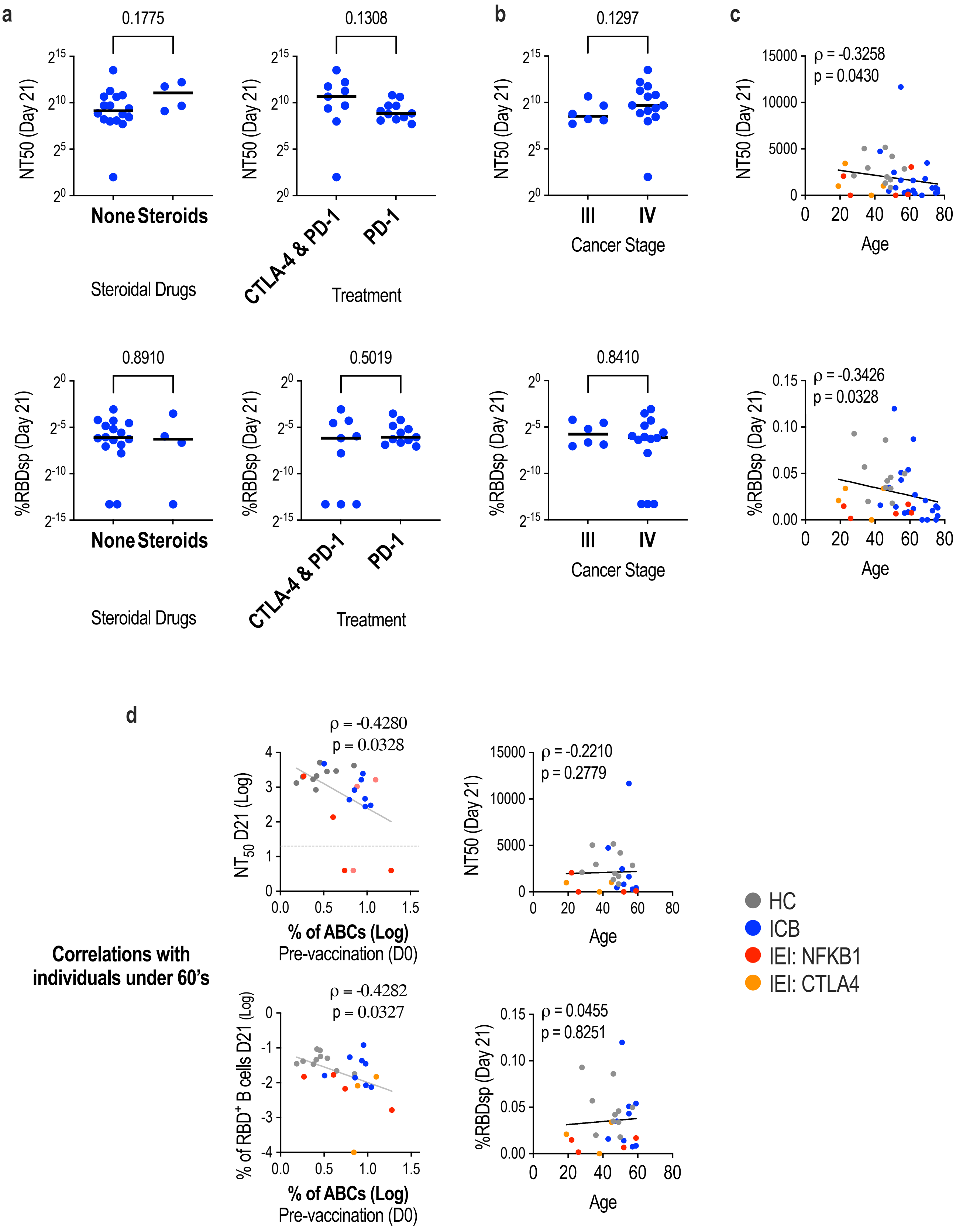

Supplementary Figure 10. Humoral response analysis based on treatment, cancer stage and age. a. Neutralising antibody titres at 50% inhibition (NT_{50}) against wildtype SARS-CoV-2 (top) and frequencies of RBD-specific B cells amongst all $CD19^+$ B cells (bottom) at day 21 after 2nd vaccine dose in cancer patients grouped by steroidal drugs treatment and ICB therapy. None=15, Steroids=4. CTLA-4 & PD-1=8, PD-1=11. **b.** Neutralising antibody titres at 50% inhibition (NT_{50}) against wildtype SARS-CoV-2 (top) and frequencies of RBD-specific B cells amongst all $CD19^+$ B cells (bottom) at day 21 after 2nd vaccine dose in cancer patients grouped by cancer stage. Stage III=6, Stage IV=13. Each dot represents a single individual. **c.** Spearman's correlations between NT_{50}s (top) or frequencies of RBD-specific B cells amongst all $CD19^+$ B cells (bottom) at day 21 and age of all individuals. Two-tailed Spearman's rank correlation coefficients (rho) and p values are shown, together with indicative linear regression lines. **d.** Spearman's correlations of ABC frequency at day 0 (left) and NT_{50}s (top) or frequencies of RBD-specific B cells amongst all $CD19^+$ B cells (bottom) at day 21 in individuals under the age of 60. Spearman's correlations of age of individuals under 60's (right) and NT_{50}s (top) or frequencies of RBD-specific B cells amongst all $CD19^+$ B cells (bottom) at day 21 after 2nd vaccine dose. Two-tailed Spearman's rank correlation coefficients (rho) and p values are shown, together with indicative linear regression lines. Each dot represents a single individual (healthy controls (HC), grey; patients with rare inborn errors of immunity (IEI): (NFKB1) red, (CTLA-4 and unclassified) orange; patients treated with ICB, blue).

Supplementary Figure 11.

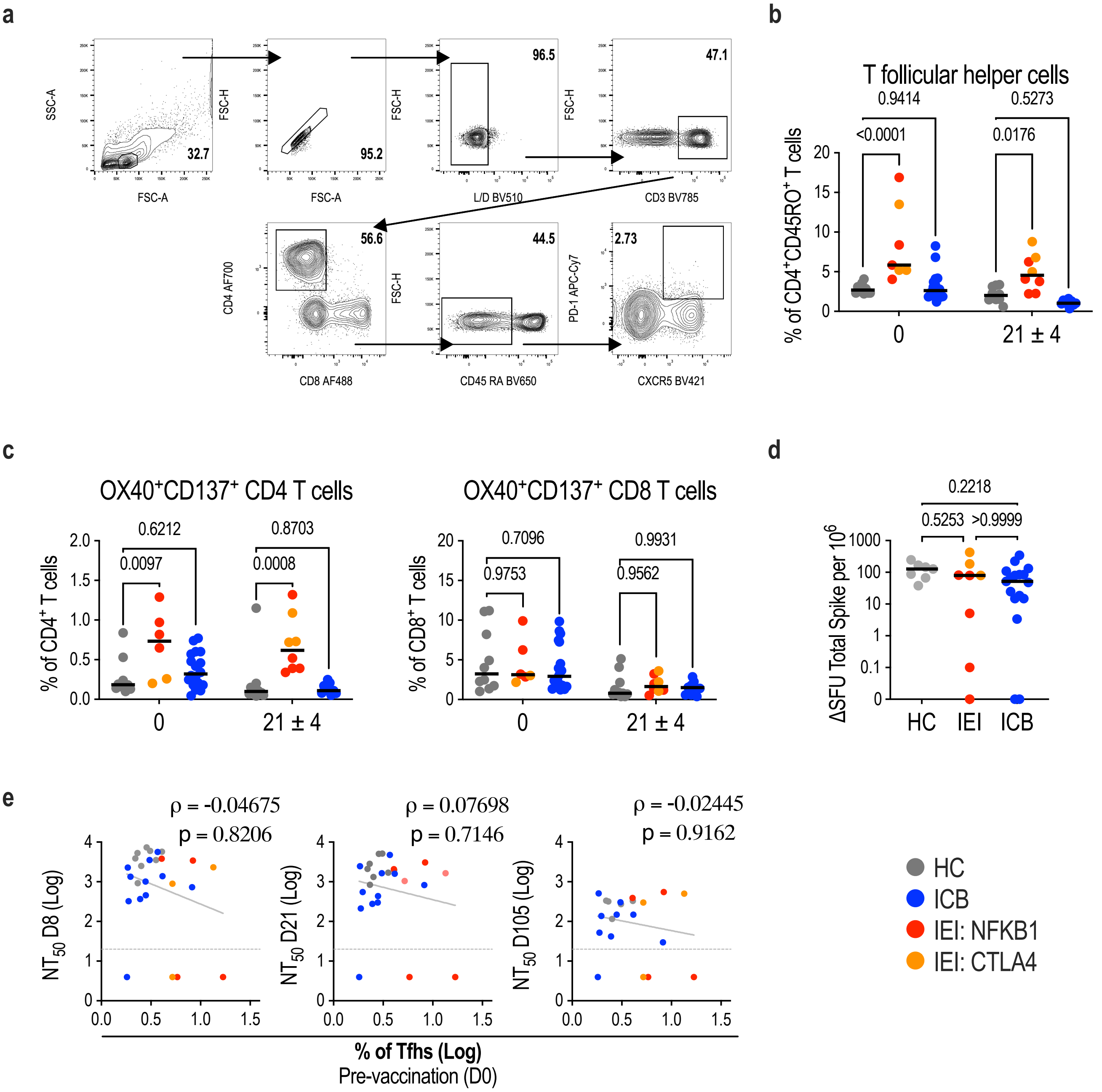

Supplementary Figure 11. T cell assessment. a. Gating strategy to identify T follicular helper cells (Tfhs, SingletsLiveCD3+CD8-CD4+CD45RA-PD-1+CXCR5+) subset. **b.** Frequencies of Tfhs amongst CD4+CD45RO+(CD45RA-) T cells in healthy controls and patients at days 0 and 21 after the second BNT162b2 dose. HC, D0=8, D21=10. IEI, D0=7, D21=8. ICB, D0=19, D21=9. **c.** Frequencies of OX40 and CD137 expression on CD4+ and CD8+ T cells amongst total CD4+ or CD8+ T cells, respectively, in healthy controls and patients at days 0 and 21 after the second BNT162b2 dose. HC, D0=8, D21=10. IEI, D0=6, D21=8. ICB, D0=18, D21=9. **d.** Spike-specific T cell responses quantified by Elispot at day 105 after the second BNT162b2 dose. SFU = Interferon gamma spot forming units. HC=7, IEI =8, ICB=16. **e.** Spearman's correlation between Tfh frequency at day 0 and NT_{50} at days 8, 21 and 105 post-second dose vaccination. Two-tailed Spearman's rank correlation coefficients (rho) and p values are shown, together with indicative linear regression line. Each dot represents a single individual (healthy controls (HC), grey; patients with rare inborn errors of immunity (IEI): (NFKB1) red, (CTLA-4 and unclassified) orange; patients treated with ICB, blue). One-way ANOVA (d) or Two-way ANOVA with Tukey's multiple comparisons test (b-c) for statistical analysis. P values are indicated.

Supplementary Figure 12.

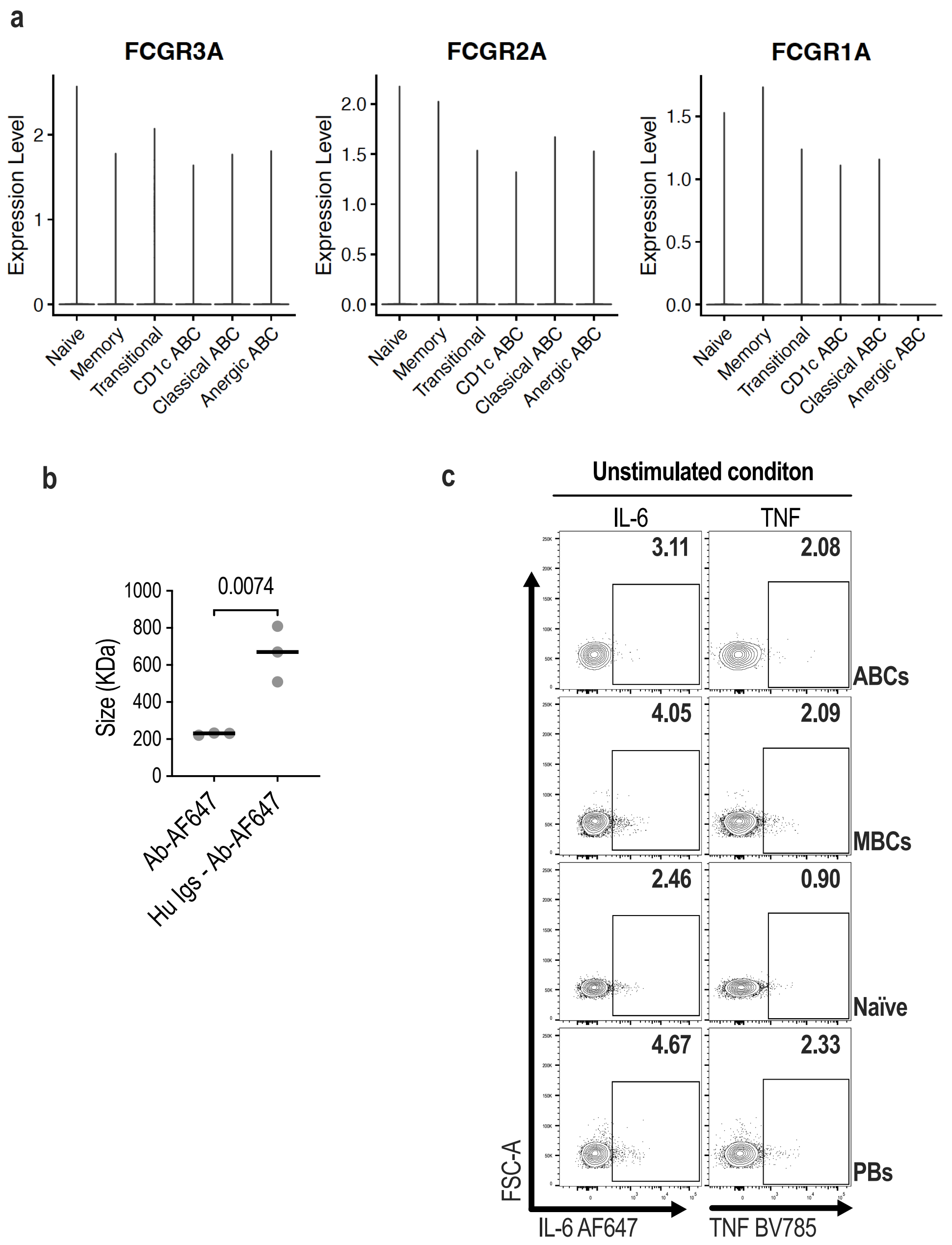

Supplementary Figure 12. Fc γ receptors expression on B cells. a. Violin plots displaying the expression levels of *FCGR3A*, *FCGR2A* and *FCGR1A* on different B cell subsets. **b.** Relative size of detection antibody (Ab-AF647) or immune complexes (Hu Igs – Ab-AF647) by means of hydrodynamic radius (R_h) measured by Microfluidic Diffusional Sizing (MDS). n=3 independent replicates per condition. **c.** Representative flow cytometry contour plots displaying cytokines production (IL-6 and TNF) by age-associated B cells (ABCs), memory B cells (MBCs), naïve B cells and plasmablasts (PBs) on unstimulated cells incubated for 5h at 37C with brefeldin A.

Supplementary Table 1.

		Table S1. Top 20 biological processes from the gene ontology enrichment analysis		
		Gene ontology terms	Fold enrichment	-LOG10(P values)
**1**	Associated to antigen presentation	**antigen processing and presentation of peptide or polysaccharide antigen via MHC class II (GO:0002504)**	28.21	1.16E+01
**2**	Associated to antigen presentation	**antigen processing and presentation of exogenous peptide antigen via MHC class II (GO:0019886)**	29.84	1.13E+01
**3**	Associated to antigen presentation	**antigen processing and presentation of peptide antigen via MHC class II (GO:0002495)**	27.97	1.10E+01
**4**	Associated to antigen presentation	**MHC class II protein complex assembly (GO:0002399)**	39.96	8.87E+00
**5**	Associated to antigen presentation	**peptide antigen assembly with MHC class II protein complex (GO:0002503)**	39.96	8.85E+00
**6**	Associated to antigen presentation	**MHC protein complex assembly (GO:0002396)**	31.97	8.24E+00
**7**	Associated to antigen presentation	**peptide antigen assembly with MHC protein complex (GO:0002501)**	31.97	8.23E+00
8	Other biological processes	regulation of neutrophil activation (GO:1902563)	21.31	3.12E+00
9	Other biological processes	regulation of PERK-mediated unfolded protein response (GO:1903897)	23.25	2.46E+00
**10**	Associated to antigen presentation	**antigen processing and presentation of exogenous peptide antigen via MHC class I, TAP-independent (GO:0002480)**	47.96	2.24E+00
11	Other biological processes	response to corticotropin-releasing hormone (GO:0043435)	38.36	2.08E+00
12	Other biological processes	positive regulation of cyclic-nucleotide phosphodiesterase activity (GO:0051343)	38.36	2.08E+00
13	Other biological processes	cellular response to corticotropin-releasing hormone stimulus (GO:0071376)	38.36	2.08E+00
14	Other biological processes	negative regulation of neutrophil activation (GO:1902564)	38.36	2.08E+00
15	Other biological processes	protection from natural killer cell mediated cytotoxicity (GO:0042270)	31.97	1.94E+00
16	Other biological processes	cellular response to laminar fluid shear stress (GO:0071499)	23.98	1.72E+00
17	Other biological processes	regulation of neutrophil degranulation (GO:0043313)	23.98	1.72E+00
**18**	Associated to antigen presentation	**antigen processing and presentation of endogenous peptide antigen via MHC class I via ER pathway, TAP-independent (GO:0002486)**	23.98	1.72E+00
**19**	Associated to antigen presentation	**antigen processing and presentation of endogenous peptide antigen via MHC class I via ER pathway (GO:0002484)**	23.98	1.72E+00
**20**	Associated to antigen presentation	**antigen processing and presentation of endogenous peptide antigen via MHC class II (GO:0002491)**	63.94	1.39E+00

Supplementary Table 1 – Top 20 biological processes from the gene ontology enrichment analysis. Statistical testing via Fisher's Exact test with correction for false discovery rate.

Supplementary Table 2.

Table S2. AIRE target genes related to Fig. 2d and Supp. Fig. 5							
1	CISH	20	MAP3K15	39	ATP2B4	57	TET2
2	GBP7	21	TP73	40	SEMA6D	58	ARHGAP19
3	PVR	22	TFCP2L1	41	SIGLEC6	59	CD3E
4	DDX60	23	INSYN2B	42	HLA-G	60	CD8A
5	PARP12	24	FNIP1	43	CDH17	61	RAG1
6	LAMP3	25	RAPGEF4	44	VWA3B	62	NLRC5
7	HLA-F	26	SYN3	45	GGN	63	SLFN12L
8	VASN	27	GRIK2	46	ZBTB32	64	ZBP1
9	SOCS3	28	ACSBG1	47	NEK2	65	PHF11
10	LIF	29	HSPH1	48	GAS2L3	66	OAS2
11	AHR	30	EML5	49	TDG	67	MX1
12	HK2	31	ST8SIA1	50	FLT3	68	CASTOR1
13	SLC24A1	32	ZBTB18	51	SCIMP	69	IL2RB
14	STXBP1	33	DBNDD1	52	PIK3R6	70	CTSW
15	STARD9	34	RANBP17	53	ADGRG5	71	RUNX2
16	OBSCN	35	ATP10A	54	RSAD2	72	SLFN12
17	SSPN	36	SEMA6A	55	HLA-E	73	CSF2RB
18	STRIP2	37	RPRM	56	STK38L	74	SOCS2
19	SLC5A3	38	PKD2				

Supplementary Table 2 – AIRE target genes related to Fig. 2d and Supp. Fig. 5

Supplementary Table 3.

Table S3. Characteristics of Inborn Errors of Immunity (IEI) patients and Immune Checkpoint blockade treated (ICB) patients

Condition	Sex	Current Treatment	Most recent IgG (6-16 g/L)	NT_{50}(D21)	% ABCs
**Healthy Controls (Age range: 31-57y)**					
Healthy Controls (n=10)	F (5), M(5)	N/A	N/A	844-5184	1.53-7.1
**Inborn Errors of Immunity (IEI) Patients (Age range: 19-61y)**					
CTLA4 haploinsufficiency (c.380A>G p.(Tyr127Cys)	F	Ig Replacement	9.56	1040	7.7
CTLA4 haploinsufficiency (c.380A>G p.(Tyr127Cys)	F	Ig Replacement	3.45	1640	12.6
LRBA compound heterozygote (c.1896C>T p.R633X) and (c.2258+4dupA p.V723_K753del)	F	Ig Replacement	11.1	4	6.93
NFKB1: c.1423delG, p.(Ala475ProfsTer10)	M	Ig Replacement	19.39	4	5.52
NFKB1: c.160-1G>A	F	None	12.62	2084	1.86
NFKB1: c.1901dupT p.(Leu636ThrfsTer11)	F	Ig Replacement	9.16	4	19
NFKB1c.1423delG, p.(Ala475ProfsTer10)	M	None	4.55	138	4.06
NFKB1: c.1190dupG p.(T398H fsTer9)	M	None	8.05	3074	4.42
No causative mutation found	M	Ig Replacement	1.4	NS	19.3
**Immune Checkpoint Blockade (ICB) Treated Patients (Age range: 43-76y)**					
Stage IIIA Oesophageal cancer	M	Ipilimumab Nivolumab	N/A	1636	8.62
Stage IIIC Melanoma	M	Pembrolizumab	N/A	300	11
Stage IV Metastatic Melanoma	M	Pembrolizumab	N/A	554	10.6
Stage IV Metastatic Renal	F	Ipilimumab Nivolumab	N/A	824	7.17
Stage IV Metastatic Renal	M	Ipilimumab* Nivolumab	N/A	4	31.9
Stage IIIB Melanoma	F	Pembrolizumab	N/A	212	7.21
Stage IV Metastatic Melanoma	M	Pembrolizumab / Ipilimumab Nivolumab	N/A	4736	3.2
Stage IIIC Melanoma	M	Pembrolizumab	N/A	276	9.57
Stage IIIB Melanoma	F	Pembrolizumab	N/A	440	6.25
Stage IV Metastatic Melanoma	M	Ipilimumab Nivolumab	N/A	2488	8.99
Stage IV Metastatic Melanoma	M	Pembrolizumab	N/A	1602	3.07
Stage IV Metastatic Melanoma	M	Pembrolizumab	N/A	669.8	11.7
Stage IV Metastatic Melanoma	M	Pembrolizumab	N/A	256.4	8.73
Stage IV Melanoma	M	Nivolumab	N/A	2100	5.48
Stage IIIC Melanoma	M	Pembrolizumab	N/A	813.2	4.98
Stage IV Metastatic Melanoma	M	Pembrolizumab	N/A	835.2	3.72
Stage IV Melanoma	F	Nivolumab	N/A	351	19.5
Stage IV Metastatic Renal	M	Ipilimumab Nivolumab	N/A	NS	22.6
Stage IV Metastatic Melanoma	F	Pembrolizumab	N/A	464	9.47

IgG, Immunoglobulin G; NT_{50}, Neutralisation titres; ABCs, Aged-associated B cells; NS, No sample collection

*Patient was off of this treatment at the time of vaccination and sampling

Supplementary Table 3 – Characteristics of Inborn Errors of Immunity (IEI) patients and Immune Checkpoint blockade treated (ICB) patients

Supplementary Table 4.

Table S4. Flow cytometry antibodies details

B Cell Panel	Source	Identifier	Clone	Lot	Concentration /Dilution
Mouse monoclonal anti-human CD11c Brilliant Violet 421	BioLegend	337226	Bu15	B279604	2 µl/test
Mouse monoclonal anti-human CD11c APC/Fire 750	BioLegend	371510	S-HCL-3	B311740	2 µl/test
Mouse monoclonal anti-human CD71 Brilliant Violet 650	BioLegend	334116	CY1G4	B322913	2 µl/test
Mouse monoclonal anti-human CD138 Brilliant Violet 711	BioLegend	356522	MI15	B312473	
Mouse monoclonal anti-human IgM Brilliant Violet 786	BioLegend	314543	MHM-88	B311995	1 µl/test
Mouse monoclonal anti-human CD19 Brilliant Ultraviolet 395	BD Biosciences	740287	HIB19	1109150	2 µl/test
Mouse monoclonal anti-human CD19 PE/Dazzle 594	BioLegend	302252	HIB19	B358141	2 µl/test
Mouse monoclonal anti-human CD38 Brilliant Ultraviolet 737	BD Biosciences	612824	HB7	1019663	0.5 µl/test
RBD tetramer Alex Fluor 488	In House	N/A	N/A	N/A	1 µg/2E6 cells
Mouse monoclonal anti-human CD85j PerCP/Cyanine 5.5	BioLegend	333714	GHI/75	B265059	2 µl/test
Mouse monoclonal anti-human IgG PE	Miltenyi Biotec	130-119-878	IS11-3B2.2.3	5210209105	1 µl/test
Mouse monoclonal anti-human IgA PE-Vio615	Miltenyi Biotec	130-116-882	REA1014	5210401106	1 µl/test
Mouse monoclonal anti-human IgD PE/Vio770	Miltenyi Biotec	130-098-583	IgD26	5210304081	5 µl/test
RBD tetramer Alexa Fluor 647	In House	N/A	N/A	N/A	1 µg/2E6 cells
Mouse monoclonal anti-human CD21 Alexa Fluor 700	BioLegend	354918	Bu32	B310409	5 µl/test
Mouse monoclonal anti-human CD27 APC/Vio700	Miltenyi Biotec	130-113-627	M-T271	5210401101	1 µl/test
Mouse monoclonal anti-human CD27 FITC	BioLegend	356404	M-T271	B342056	1 µl/test
Mouse monoclonal anti-human CD20 PE-Vio 770	Miltenyi Biotec	130-113-375	LT20	5200706873	2 µl/test
Mouse monoclonal anti-human TNF-α Brilliant Violet 785	BioLegend	502948	MAb11	B359385	2 µl/test
Mouse monoclonal anti-human IL-6 Alexa Fluor 647	BioLegend	501124	MQ2-13A5	B381219	2 µl/test
**T Cell Panel**	**Source**	**Identifier**	**Clone**	**Lot**	**Concentration /Dilution**
Mouse monoclonal anti-human CD3 Brilliant Violet 785	BioLegend	344842	SK7	B281768	1 µl/test
Mouse monoclonal anti-human CD4 Alexa Fluor 700	BioLegend	344622	SK3	B347223	2 µl/test
Mouse monoclonal anti-human CD8a Alexa Fluor 488	BioLegend	301021	RPA-T8	B284960	2 µl/test
Mouse monoclonal anti-human CD45RA Brilliant Violet 650	BioLegend	304136	HI100	B317826	1 µl/test
Mouse monoclonal anti-human CCR7 PE-Cyanine 7	BioLegend	353226	G043H7	B305236	2 µl/test
Mouse monoclonal anti-human CD38 Brilliant Ultraviolet 737	BD Biosciences	612824	HB7	1019663	0.5 µl/test
Mouse monoclonal anti-human CD137 APC	BioLegend	309810	4B4-1	B320971	2 µl/test
Mouse monoclonal anti-human OX40 PE	BioLegend	350005	Ber-ACT35	B277962	2 µl/test
Mouse monoclonal anti-human CXCR5 Brilliant Violet 421	BioLegend	356920	J252D4	B325837	2 µl/test
Armenian Hamster monoclonal anti-human ICOS Brilliant Violet 711	BioLegend	313548	C398.4A	B328289	2 µl/test
Mouse monoclonal anti-human PD-1 APC/Cyanine 7	BioLegend	367416	29F.1A12	B280485	2 µl/test
**Others**	**Source**	**Identifier**	**Clone**	**Lot**	**Concentration /Dilution**
TruStain (Fc Block)	BioLegend	422302	N/A	B335814	5 µl/test
Aquafluor Brilliant Violet 405	Invitrogen	L34966A	N/A	2268307	1µl/500 µl

N/A= Not applicable

Supplementary Table 4 – Flow cytometry antibodies details

CITIID-NIHR BioResource COVID-19 BioResource Collaboration

Stephen Baker[2,6], John Bradley[1,3,6,11,15] , Patrick Chinnery[3,23,24], Daniel Cooper[11, 25], Gordon Dougan[2,6], Ian Goodfellow[7], Ravindra Gupta[2,6,13,16], Nathalie Kingston[3,4], Paul J. Lehner[2,6,13], Paul A. Lyons[2,6], Nicholas J. Matheson[2,6,13,33], Caroline Saunders[9], Kenneth G. C. Smith[2,6], Charlotte Summers[6,12,26], James Thaventhiran[19], M. Estee Torok [6,13,14], Mark R. Toshner[6,8,26], Michael P. Weekes[2,6,13,34], Gisele Alvio[9], Sharon Baker[9], Areti Bermperi[9], Karen Brookes[9], Ashlea Bucke, Jo Calder, Laura Canna, Cherry Crucusio, Isabel Cruz[9], Ranalie de Jesus[9], Katie Dempsey[9], Giovanni Di Stephano[9], Jason Domingo[9], Anne Elmer[9], Julie Harris, Sarah Hewitt, Heather Jones[9], Sherly Jose[9], Jane Kennet, Yvonne King, , Jenny Kourampa[9], Emily Li, Caroline McMahon[9], Anne Meadows, Vivien Mendoza[9], Criona O'Brien, Charmain Ocaya[9], Ciro Pasquale[9], Marlyn Perales[9], Jane Price, Rebecca Rastall, Carla Ribeiro[9], Jane Rowlands, Valentina Ruffolo, Hugo Tordesillas, Phoebe Vargas[9], Bensi Vergese[9], Laura Watson[9], Jieniean Worsley[9], Julie-Ann Zerrudo[9], Laura Bergamashi[2,6], Ariana Betancourt, Georgie Bower, Ben Bullman, Chiara Cossetti, Aloka De Sa, Benjamin J. Dunmore, Maddie Epping, Stuart Fawke, Stefan Gräf [3,6], Richard Grenfell, Andrew Hinch, Josh Hodgson, Christopher Huang, Oisin Huhn, Kelvin Hunter[2,6], Isobel Jarvis, Emma Jones, Maša Josipović, Ekaterina Legchenko, Daniel Lewis, Joe Marsden, Jennifer Martin, Federica Mescia[2,6], Ciara O'Donnell, Ommar Omarjee, Marianne Perera, Linda Pointon, Nicole Pond, Nathan Richoz, Nika Romashova, Natalia Savoinykh, Rahul Sharma, Joy Shih, Mateusz Strezlecki, Rachel Sutcliffe, Tobias Tilly, Zhen Tong, Carmen Treacy, Lori Turner, Jennifer Wood, Marta Wylot, John Allison[3,4], Heather Biggs[3,18], John R. Bradley[1,3,6,11,15], Helen Butcher[3,5], Daniela Caputo[3,5], Matt Chandler[3,5], Patrick Chinnery[3,23,24], Debbie Clapham-Riley[3,5], Eleanor Dewhurst[3,5], Christian Fernandez[3,4,] Anita Furlong[3,5], Barbara Graves[3,5], Jennifer Gray[3,5], Sabine Hein[3,5], Tasmin Ivers[3,5], Emma Le Gresley[3,5], Rachel Linger[3,5], Mary Kasanicki[3,11], Rebecca King[3,5], Sarah Meloy[3,5], Alexei Moulton[3,5], Francesca Muldoon[3,5], Nigel Ovington[3,4], Sofia Papadia[3,5], Christopher J. Penkett[3,4], Isabel Phelan[3,5], Venkatesh Ranganath[3,4], Roxana Paraschiv[3,4], Abigail Sage[3,5], Jennifer Sambrook[3,4], Ingrid Scholtes[3,5], Katherine Schon[3,17,18], Hannah Stark[3,5], Kathleen E. Stirrups[3,4], Paul Townsend[3,4], Neil Walker[3,4], Jennifer Webster[3,5], Mayurun Selvan[35], Petra, Polgarova[12],Sarah L. Caddy[2,6], Laura G. Caller[20,21], Yasmin Chaudhry[7], Martin D. Curran[22], Theresa Feltwell[6], Stewart Fuller[20], Iliana Georgana[7], Grant Hall[7], William L. Hamilton[6,13,14], Myra Hosmillo[7], Charlotte J. Houldcroft[6], Rhys Izuagbe[7], Aminu S. Jahun[7], Fahad A. Khokhar[2,6], Anna G. Kovalenko[7], Luke W. Meredith[7], Surendra Parmar[22], Malte L. Pinckert[7], Anna Yakovleva[7], Emily C. Horner[19], Lucy Booth[19], Alexander Ferreira[19], Rebecca Boston[19], Robert Hughes[19], Juan Carlos Yam-Puc[19], Nonantzin Beristain-Covarrubias[19], Maria Rust[19], Thevinya Gurugama[19], Lihinya Gurugama[19], Thomas E. Mulroney[19], Sarah Spencer[19], Zhaleh Hosseini[19], Kate Williamson[19].

[1]NIHR Cambridge Biomedical Research Centre, Cambridge Biomedical Campus, Cambridge, UK
[2]Cambridge Institute of Therapeutic Immunology and Infectious Disease (CITIID), Jeffrey Cheah Biomedical Centre, Cambridge Biomedical Campus, Cambridge, UK
[3]NIHR BioResource, Cambridge University Hospitals NHS Foundation Trust, Cambridge, UK
[4]Department of Haematology, School of Clinical Medicine, University of Cambridge, Cambridge Biomedical Campus, Cambridge, UK
[5]Department of Public Health and Primary Care, School of Clinical Medicine, University of Cambridge, Cambridge Biomedical Campus, Cambridge, UK
[6]Department of Medicine, School of Clinical Medicine, University of Cambridge, Cambridge Biomedical Campus, Cambridge, UK
[7]Division of Virology, Department of Pathology, University of Cambridge, Cambridge, UK
[8]Royal Papworth Hospital NHS Foundation Trust, Cambridge, UK
[9]Cambridge Clinical Research Centre, Addenbrooke's Hospital, Cambridge University Hospitals NHS Foundation Trust, Cambridge, UK
[10]Intensive Care Unit, Royal Papworth Hospital NHS Foundation Trust, Cambridge, UK
[11]Addenbrooke's Hospital, Cambridge University Hospitals NHS Foundation Trust, Cambridge Biomedical Campus, Cambridge, UK
[12]Intensive Care Unit, Addenbrooke's Hospital, Cambridge University Hospitals NHS Foundation Trust, Cambridge Biomedical Campus, Cambridge, UK
[13]Department of Infectious Diseases, Addenbrooke's Hospital, Cambridge University NHS Hospitals Foundation Trust, Cambridge, UK
[14]Department of Microbiology, Addenbrooke's Hospital, Cambridge University NHS Hospitals Foundation Trust, Cambridge, UK
[15]Department of Renal Medicine, Addenbrooke's Hospital, Cambridge University Hospitals NHS Foundation Trust, Cambridge, UK
[16]Africa Health Research Institute, Durban, South Africa
[17]Clinical Genetics, Addenbrooke's Hospital, Cambridge University Hospitals NHS Foundation Trust, Cambridge, UK
[18]Department of Clinical Neurosciences, School of Clinical Medicine, University of Cambridge, Cambridge Biomedical Campus, Cambridge, UK
[19]MRC Toxicology Unit, Gleeson Building, Tennis Court Road, Cambridge, UK
[20]University of Cambridge, Cambridge, UK
[21]The Francis Crick Institute, London, UK
[22]Public Health England, Clinical Microbiology and Public Health Laboratory, Cambridge, UK
[23]Department of Clinical Neurosciences, School of Clinical Medicine, University of Cambridge, Cambridge Biomedical Campus, Cambridge, UK
[24]Medical Research Council Mitochondrial Biology Unit, Cambridge Biomedical Campus, Cambridge, UK
[25]Global and Tropical Health Division, Menzies School of Heath Research and Charles Darwin University, Darwin, Northern Territory, Australia
[26]Heart and Lung Research Institute, Cambridge Biomedical Campus, Cambridge, UK
[27]Department of Rheumatology, Addenbrooke's Hospital, Cambridge University Hospitals NHS Foundation Trust, Cambridge, UK
[28]Cambridge Cancer Trials Centre, Addenbrooke's Hospital, Cambridge University Hospitals NHS Foundation Trust, Cambridge, UK
[29]Department of Paediatrics, University of Cambridge, Cambridge Biomedical Campus, Cambridge, UK
[30]Patient Safety, Addenbrooke's Hospital, Cambridge University Hospitals NHS Foundation Trust, Cambridge, UK
[31]Clinical Research Network: Eastern, Addenbrooke's Hospital, Cambridge University Hospitals NHS Foundation Trust, Cambridge, UK
[32]Institute of Metabolic Science, Addenbrooke's Hospital, Cambridge University Hospitals NHS Foundation Trust, Cambridge, UK
[33]NHS Blood and Transplant, Cambridge, UK
[34]Cambridge Institute for Medical Research, Biomedical Campus, Hills Rd, Cambridge UK
[35] Department of Respiratory Medicine, Cambridge University Hospitals NHS Foundation Trust, Cambridge, UK
