# Supplementary material for: Age-associated B cells predict impaired humoral immunity after COVID-19 vaccination in patients receiving immune checkpoint blockade
Source: Nat Commun. 2023 Jun 27;14:3292. doi: 10.1038/s41467-023-38810-0 (PMC10299999; doi:10.1038/s41467-023-38810-0)
Supplement: Supplementary file 3 — Description of Additional Supplementary Files [file 41467_2023_38810_MOESM3_ESM.pdf]

Description of Additional Supplementary Files

File Name: Supplementary Data 1

Description: Differentially expressed genes related to Figure 1e. Statistical testing via Wilcoxon rank sum test with Bonferroni correction

File Name: Supplementary Data 2

Description: Differentially expressed genes related to Figure 2a. Statistical testing via Wilcoxon rank sum test with Bonferroni correction.
