# Supplementary material for: Age-associated B cells predict impaired humoral immunity after COVID-19 vaccination in patients receiving immune checkpoint blockade
Source: Nat Commun. 2023 Jun 27;14:3292. doi: 10.1038/s41467-023-38810-0 (PMC10299999; doi:10.1038/s41467-023-38810-0)
Supplement: Supplementary file 6 — Reporting Summary [file 41467_2023_38810_MOESM6_ESM.pdf]

nature portfolio

Corresponding author(s): James E. D. Thaventhiran
Juan Carlos Yam-Puc

Last updated by author(s): 2023/04/04

Reporting Summary

Nature Portfolio wishes to improve the reproducibility of the work that we publish. This form provides structure for consistency and transparency in reporting. For further information on Nature Portfolio policies, see our Editorial Policies and the Editorial Policy Checklist.

Statistics

For all statistical analyses, confirm that the following items are present in the figure legend, table legend, main text, or Methods section.

n/a	Confirmed	
☐	☒	The exact sample size (*n*) for each experimental group/condition, given as a discrete number and unit of measurement
☐	☒	A statement on whether measurements were taken from distinct samples or whether the same sample was measured repeatedly
☐	☒	The statistical test(s) used AND whether they are one- or two-sided<br>*Only common tests should be described solely by name; describe more complex techniques in the Methods section.*
☒	☐	A description of all covariates tested
☐	☒	A description of any assumptions or corrections, such as tests of normality and adjustment for multiple comparisons
☐	☒	A full description of the statistical parameters including central tendency (e.g. means) or other basic estimates (e.g. regression coefficient) AND variation (e.g. standard deviation) or associated estimates of uncertainty (e.g. confidence intervals)
☐	☒	For null hypothesis testing, the test statistic (e.g. *F*, *t*, *r*) with confidence intervals, effect sizes, degrees of freedom and *P* value noted<br>*Give P values as exact values whenever suitable.*
☒	☐	For Bayesian analysis, information on the choice of priors and Markov chain Monte Carlo settings
☒	☐	For hierarchical and complex designs, identification of the appropriate level for tests and full reporting of outcomes
☒	☐	Estimates of effect sizes (e.g. Cohen's *d*, Pearson's *r*), indicating how they were calculated

Our web collection on statistics for biologists contains articles on many of the points above.

Software and code

Policy information about availability of computer code

Data collection	CellRanger<br>FACSDiva
Data analysis	Single cell transcriptome and VDJ data was analysed using Cell Ranger software version 6.1.1 and 7.0.1 respectively, citeseq version 1.5.0, R version 4.0.3 and R packages (Seurat 4.1.0, SeuratObject 4.0.4, Scuttle 1.0.4, rstatix 0.7.0, tidyverse 1.3.0 and dplyr 1.0.8) and python packages (dandelion 0.3.0, pandas 1.1.3, numpy 1.19.2, networkx 2.5, scipy 1.5.2, blast 2.11.0, igblast 1.17.1, anndata 0.7.6, scanpy 1.7.2, changeo 1.0.2 and airr 1.3.1). Flow cytometry data and antibody titres were analysed using FlowJo 10.7.2 and GraphPad Prism 9.1.2 (225), respectively. FCS files were analysed using FlowJo 10.7.2. Additionally, a uniform manifold approximation and projection (UMAP) algorithm for dimensional reduction was performed on a concatenated FCS file comprising events from the CD19+ gate, utilizing the UMAP FlowJo plugin (v3.1). XShift (v1.4.1) and ClusterExplorer (1.5.15) plugins were used for unsupervised cluster generation and exploration, respectively. Figures were produced using ggplot2 3.3.5, gridExtra 2.3, ComplexHeatmap 2.6.2, Nebulosa 1.0.2, RColorBrewer 1.1-2, ggrepel 0.9.1, ggpubr 0.4.0, scales 1.1.1, showtext 0.9-5 in R and GraphPad Prism 9.1.2 (225) and FlowJo 10.7.1.

For manuscripts utilizing custom algorithms or software that are central to the research but not yet described in published literature, software must be made available to editors and reviewers. We strongly encourage code deposition in a community repository (e.g. GitHub). See the Nature Portfolio guidelines for submitting code & software for further information.

Data

Policy information about availability of data

All manuscripts must include a data availability statement. This statement should provide the following information, where applicable:

- Accession codes, unique identifiers, or web links for publicly available datasets
- A description of any restrictions on data availability
- For clinical datasets or third party data, please ensure that the statement adheres to our policy

The raw and processed sequencing data generated in this study have been deposited in the Gene Expression Omnibus (GEO) database under accession code GSE207475 (https://www.ncbi.nlm.nih.gov/geo/query/acc.cgi?acc=GSE207475). The SLE data used in this study are available in the GEO database under accession code GSE163121 (https://www.ncbi.nlm.nih.gov/geo/query/acc.cgi?acc=GSE163121). Gene expression data in different immune cells and different tissues are extracted from the human protein atlas website (https://v22.proteinatlas.org/download/rna_immune_cell.tsv.zip and https://v22.proteinatlas.org/download/rna_tissue_consensus.tsv.zip, respectively). GRCh38 genome and GRCh38 VDJ genome are downloaded from https://cf.10xgenomics.com/supp/cell-exp/refdata-gex-GRCh38-2020-A.tar.gz and https://cf.10xgenomics.com/supp/cell-vdj/refdata-cellranger-vdj-GRCh38-alts-ensembl-7.0.0.tar.gz, respectively. All scripts used for processing the sequencing data and generating figures in this work are deposited in Zenodo (https://doi.org/10.5281/zenodo.7806635). Source data are provided with this paper.

Human research participants

Policy information about studies involving human research participants and Sex and Gender in Research.

Reporting on sex and gender	Information on sex was obtained for all participants (Supplementary Table 3). Where sex may influence the results obtained, a comprehensive analysis disaggregated for biological sex is provided in Fig. 1c, Supplementary Fig. 1e-f, Supplementary Fig. 8a.
Population characteristics	Information about the individuals enrolled in this study is provided in Supplementary Table 3: age range, sex, disease state and diagnosis and treatment.
Recruitment	Enrolment of patients into this study was based on the deficiency of particular genes of interest (IEI: CTLA4, LRBA, NFKB1, NFKB2) or clinical diagnostics (ICB, n = 19), whilst healthy controls for this study were enrolled based on their clinical healthy status (n = 10).
Ethics oversight	The research was conducted in accordance with the principles of Good Clinical Practice and following approved protocols of the NIHR National Bioresource. Samples were collected with the written informed consent of all study participants under the NIHR National BioResource - Research Tissue Bank (NBR-RTB) ethics (REC:17/EE/0025) or under the Barts and the London Immunology Registry (REC: 11/LO/1689). Clinical data were collected by Clinical Immunology Consultants at Cambridge University Hospital and Bart's Health via the Electronic Healthcare Record (Epic), or direct patient contact.<br>The patients and healthy controls were consented under the East of England Cambridge South national research ethics committee (REC) reference 13/EE/0325 or Barts and the London Immunology Registry (REC: 11/LO/1689).

Note that full information on the approval of the study protocol must also be provided in the manuscript.

Field-specific reporting

Please select the one below that is the best fit for your research. If you are not sure, read the appropriate sections before making your selection.

☒ Life sciences ☐ Behavioural & social sciences ☐ Ecological, evolutionary & environmental sciences

For a reference copy of the document with all sections, see nature.com/documents/nr-reporting-summary-flat.pdf

Life sciences study design

All studies must disclose on these points even when the disclosure is negative.

Sample size	Enrolment of patients into this study was based on the deficiency of particular genes of interest (IEI: CTLA4, LRBA, NFkB1, NFkB2) or clinical diagnostics (ICB, n = 19), whilst healthy controls for this study were enrolled based on their clinical healthy status (n = 10). Sample-size calculation was limited due to IEI patients in the study (n = 9) reflect a rare population of individuals within the general population that we were able to recruit.
Data exclusions	Samples with evidence of previous SARS-CoV-2 infection may be excluded.
Replication	Multiple independent samples were tested at all time points as indicated in the figures, figure legends and methods.
Randomization	Non-interventional study. Not applicable

Blinding	Non-interventional study. Not applicable

Reporting for specific materials, systems and methods

We require information from authors about some types of materials, experimental systems and methods used in many studies. Here, indicate whether each material, system or method listed is relevant to your study. If you are not sure if a list item applies to your research, read the appropriate section before selecting a response.

Materials & experimental systems

n/a	Involved in the study
☐	☒ Antibodies
☐	☒ Eukaryotic cell lines
☒	☐ Palaeontology and archaeology
☒	☐ Animals and other organisms
☒	☐ Clinical data
☒	☐ Dual use research of concern

Methods

n/a	Involved in the study
☒	☐ ChIP-seq
☐	☒ Flow cytometry
☒	☐ MRI-based neuroimaging

Antibodies

Antibodies used

Antibodies used in this study are detailed in Supplementary Table 4.

Validation

Biolegend
All of our products undergo industry-leading rigorous quality control (QC) testing to ensure the highest level of performance and reproducible results. Each lot is compared to an internally established "gold standard" to maintain lot-to-lot consistency. We also conduct wide-scale stability studies to guarantee an accurate shelf-life for our products.Additionally, we test the majority of our products on endogenous cells rather than transfected or immortal cells that may overexpress the analyte. We assess our reagents with samples and protocols that reflect our customers' experience. Our willingness to monitor the quality of our reagents extends beyond our lab and into yours.
For Flow Cytometry reagents validation, primary antibodies specificity testing of 1-3 target cell types with either single- or multi-color analysis (including positive and negative cell types) is done. Once specificity is confirmed, each new lot must perform with similar intensity to the in-date reference lot. Brightness (MFI) is evaluated from both positive and negative populations. Each lot product is validated by QC testing with a series of titration dilutions.
Mouse monoclonal anti-human CD11c Brilliant Violet 421 337226 Bu15 B279604 2 µl/test
Mouse monoclonal anti-human CD11c APC/Fire 750 371510 S-HCL-3 B311740 2 µl/test
Mouse monoclonal anti-human CD71 Brilliant Violet 650 334116 CY1G4 B322913 2 µl/test
Mouse monoclonal anti-human CD138 Brilliant Violet 711 356522 MI15 B312473
Mouse monoclonal anti-human IgM Brilliant Violet 786 314543 MHM-88 B311995 1 µl/test
Mouse monoclonal anti-human CD19 PE/Dazzle 594 302252 HIB19 B358141 2 µl/test
Mouse monoclonal anti-human CD85j PerCP/Cyanine 5.5 333714 GHI/75 B265059 2 µl/test
Mouse monoclonal anti-human CD21 Alexa Fluor 700 354918 Bu32 B310409 5 µl/test
Mouse monoclonal anti-human CD27 FITC 356404 M-T271 B342056 1 µl/test
Mouse monoclonal anti-human TNF-α Brilliant Violet 785 502948 MAb11 B359385 2 µl/test
Mouse monoclonal anti-human IL-6 Alexa Fluor 647 501124 MQ2-13A5 B381219 2 µl/test
Mouse monoclonal anti-human CD3 Brilliant Violet 785 344842 SK7 B281768 1 µl/test
Mouse monoclonal anti-human CD4 Alexa Fluor 700 344622 SK3 B347223 2 µl/test
Mouse monoclonal anti-human CD8a Alexa Fluor 488 301021 RPA-T8 B284960 2 µl/test
Mouse monoclonal anti-human CD45RA Brilliant Violet 650 304136 HI100 B317826 1 µl/test
Mouse monoclonal anti-human CCR7 PE-Cyanine 7 353226 G043H7 B305236 2 µl/test
Mouse monoclonal anti-human CD137 APC 309810 4B4-1 B320971 2 µl/test
Mouse monoclonal anti-human OX40 PE 350005 Ber-ACT35 B277962 2 µl/test
Mouse monoclonal anti-human CXCR5 Brilliant Violet 421 356920 J252D4 B325837 2 µl/test
Armenian Hamster monoclonal anti-human ICOS Brilliant Violet 711 313548 C398.4A B328289 2 µl/test
Mouse monoclonal anti-human PD-1 APC/Cyanine 7 367416 29F.1A12 B280485 2 µl/test

BD Biosciences
BD Biosciences not only develops its own antibodies but also collaborates with research scientists around the world to license their antibodies. We provide accessibility to the flow cytometry community by conjugating antibodies to a broad portfolio of high-performing dyes, including our vastly popular portfolio of BD Horizon Brilliant" Dyes.
A world-class team of research scientists helps ensure that these reagents work reliably and consistently for flow cytometry applications. The specificity is confirmed by using multiple applications that may include a combination of flow cytometry, immunofluorescence, immunohistochemistry or western blot to test a combination of primary cells, cell lines or transfectant models. All flow cytometry reagents are titrated on the relevant positive or negative cells. To save time and cell samples for researchers, pre-titrated test size reagents are bottled at an optimal concentration, with the best signal-to-noise ratio on relevant models. You can look up the Certificate of Analysis and the concentration of test-size human reagents from specific lots via the Concentration Lookup

page or BD Regulatory Documents.
Technical data sheets provide data generated on the relevant primary model at this optimal concentration based on a titration curve.
QC data on any lot of reagent can be requested through ResearchApplications@bd.com.
We strive to ensure different production batches are consistent regardless of type of antigen or fluorochrome through exhaustive testing and strict adherence to quality control standards. Testing with prior batches as reference helps you obtain consistent results with the new batch relative to the previous batches.
Mouse monoclonal anti-human CD19 Brilliant Ultraviolet 395 740287 HIB19 1109150 2 µl/test
Mouse monoclonal anti-human CD38 Brilliant Ultraviolet 737 612824 HB7 1019663 0.5 µl/test

Miltenyi
Miltenyi provides a lot-specific certificates and material safety data sheets for their products finder, where you can enter the lot number and then you can download the certificates of how each antibody has been tested and validated.
All our antibodies are rigorously tested and validated before release. In the application section on the product page, you can find examples of typical performance data. In addition, we provide extended validation data highlighting details of antibody performance, specificity, and fixation compatibility. All antibodies for which any of these datasets are already available will be indicated with the extended validation stamp.
Specificity is validated by Epitope competition assay, the clone being used is compared with other known clones recognizing the same antigen in a competition assay. Cells are incubated with an excess of the antibody of interest followed by staining with fluorochrome-conjugated antibodies of other known clones against the same marker. Based on the fluorescence signal obtained, the clones are identified as recognizing completely overlapping (++), partially overlapping (+), or completely different epitopes (-) of the marker.
Performance comparison. Selected fluorochrome conjugated antibodies from Miltenyi Biotec are compared to commercially available hybridoma clones in flow cytometry analysis.
Fixation data. To provide an indication on how an antibody performs after fixation of cells, in-house data on staining results before and after fixation with 3.7% formaldehyde using Miltenyi Biotec antibodies are provided. Different experimental settings may lead to different results.
Mouse monoclonal anti-human IgG PE 130-119-878 IS11-3B2.2.3 5210209105 1 µl/test
Mouse monoclonal anti-human IgA PE-Vio615 130-116-882 REA1014 5210401106 1 µl/test
Mouse monoclonal anti-human IgD PE/Vio770 130-098-583 IgD26 5210304081 5 µl/test
Mouse monoclonal anti-human CD27 APC/Vio700 130-113-627 M-T271 5210401101 1 µl/test
Mouse monoclonal anti-human CD20 PE-Vio 770 130-113-375 LT20 5200706873 2 µl/test

Eukaryotic cell lines

Policy information about cell lines and Sex and Gender in Research

Cell line source(s): Luminescent HEK293T reporter cells for SARS-CoV-2 were generated in the Matheson lab as previously described (https://doi.org/10.1371/journal.ppat.1010265). They are available from the National Institute for Biological Standards and Control (NIBSC, www.nibsc.org, catalogue number 101062).

Authentication: HEK293T cells were authenticated by STR profiling as previously described (https://doi.org/10.1371/journal.ppat.1010265).

Mycoplasma contamination: Luminescent HEK293T reporter cells for SARS-CoV-2 were regularly screened and confirmed to be mycoplasma negative (Lonza MycoAlert and IDEXX BioAnalytics).

Commonly misidentified lines (See ICLAC register): No commonly misidentified lines were used.

Flow Cytometry

Plots

Confirm that:

- [x] The axis labels state the marker and fluorochrome used (e.g. CD4-FITC).
- [x] The axis scales are clearly visible. Include numbers along axes only for bottom left plot of group (a 'group' is an analysis of identical markers).
- [x] All plots are contour plots with outliers or pseudocolor plots.
- [x] A numerical value for number of cells or percentage (with statistics) is provided.

Methodology

Sample preparation: Frozen PBMCs were thawed and stained with B cell panel antibody cocktail (Supplementary Table 4).

Instrument: BD LSRFortessa

Software: All samples were acquired using FACSDIVA Software. FCS files were analysed using FlowJo 10.7.2. Additionally, a uniform manifold approximation and projection (UMAP) algorithm for dimensional reduction was performed on a concatenated FCS file comprising events from the CD19+ gate, utilizing the UMAP FlowJo plugin (v3.1). XShift (v1.4.1) and ClusterExplorer

	(1.5.15) plugins were used for unsupervised cluster generation and exploration, respectively.
Cell population abundance	We did flow cytometry without sorting. RBD-binding B cells are scarce, we acquired between 1x10^6 - 4x10^6 events per sample.
Gating strategy	Examples of gating strategies are presented in Supplementary Figures, with further strategies available upon request.

☒ Tick this box to confirm that a figure exemplifying the gating strategy is provided in the Supplementary Information.
